# A data quality assessment to inform hypertension surveillance using primary care electronic medical record data from Alberta, Canada

**DOI:** 10.1186/s12889-021-10295-w

**Published:** 2021-02-02

**Authors:** Stephanie Garies, Kerry McBrien, Hude Quan, Donna Manca, Neil Drummond, Tyler Williamson

**Affiliations:** 1 Department of Family Medicine, University of Calgary, G012 Health Sciences Centre, 3330 Hospital Drive NW, Calgary, Alberta T2N 4N1 Canada; 2 Department of Community Health Sciences, University of Calgary, 3280 Hospital Drive NW, Calgary, Alberta T2N 4Z6 Canada; 3 Department of Family Medicine, University of Alberta, 6-10 University Terrace, Edmonton, Alberta T6G 2T4 Canada; 4 School of Public Health, University of Alberta, 3-300 Edmonton Clinic Health Academy, 11405-87 Ave, Edmonton, Alberta T6G 1C9 Canada

**Keywords:** Data quality, Primary care, Electronic medical records, Hypertension, Surveillance

## Abstract

**Background:**

Hypertension is a common chronic condition affecting nearly a quarter of Canadians. Hypertension surveillance in Canada typically relies on administrative data and/or national surveys. Routinely-captured data from primary care electronic medical records (EMRs) are a complementary source for chronic disease surveillance, with longitudinal patient-level details such as sociodemographics, blood pressure, weight, prescribed medications, and behavioural risk factors. As EMR data are generated from patient care and administrative tasks, assessing data quality is essential before using for secondary purposes. This study evaluated the quality of primary care EMR data from one province in Canada within the context of hypertension surveillance.

**Methods:**

We conducted a cross-sectional, descriptive study using primary care EMR data collected by two practice-based research networks in Alberta, Canada. There were 48,377 adults identified with hypertension from 53 clinics as of June 2018. Summary statistics were used to examine the quality of data elements considered relevant for hypertension surveillance.

**Results:**

Patient year of birth and sex were complete, but other sociodemographic information (ethnicity, occupation, education) was largely incomplete and highly variable. Height, weight, body mass index and blood pressure were complete for most patients (over 90%), but a small proportion of outlying values indicate data inaccuracies were present. Most patients had a relevant laboratory test present (e.g. blood glucose/glycated hemoglobin, lipid profile), though a very small proportion of values were outside a biologically plausible range. Details of prescribed antihypertensive medication, such as start date, strength, dose, frequency, were mostly complete. Nearly 80% of patients had a smoking status recorded, though only 66% had useful information (i.e. categorized as current, past, or never), and less than half had their alcohol use described; information related to amount, frequency or duration was not available.

**Conclusions:**

Blood pressure and prescribed medications in primary care EMR data demonstrated good completeness and plausibility, and contribute valuable information for hypertension epidemiology and surveillance. The use of other clinical, laboratory, and sociodemographic variables should be used carefully due to variable completeness and suspected data errors. Additional strategies to improve these data at the point of entry and after data extraction (e.g. statistical methods) are required.

## Background

Hypertension is a common chronic condition, affecting more than one in five Canadians, and is associated with an increased risk of cardiovascular disease and mortality, as well as considerable economic and societal costs [1]. Monitoring the incidence and prevalence of hypertension over time is an important part of surveillance systems and public health activities. In Canada, administrative databases, which include in-patient hospital discharges and physician billing claims, are often used to report on hypertension prevalence estimates, such as the Canadian Chronic Disease Surveillance System (CCDSS) [2]. While administrative sources provide population-level data for those who have encountered the healthcare system, there are a lack of clinical details that are essential for better understanding the patient context and disease severity, including blood pressure (BP), body mass index (BMI), and lifestyle risk factors. Physical measures surveys are another commonly used source, as they obtain directly measured BP coupled with health-related interviews, as achieved by the Canadian Health Measures Survey (CHMS) [3]. However, these surveys are costly to maintain, response rates are often low, and the cross-sectional design does not allow for longitudinal follow-up.

A contemporary approach to hypertension surveillance is utilizing the clinically-generated, detailed data from electronic medical records (EMR), particularly from primary care settings where chronic conditions are largely diagnosed and managed [4, 5]. EMR adoption among Canadian family physicians is growing, with an estimated 83% now using EMRs in practice to some degree in 2018 [6]. Additionally, linkages between primary care EMR and administrative data can further enhance surveillance opportunities by providing a more complete perspective of disease manifestation and current management practices. Because EMR data are recorded to support individual patient care and administrative tasks, they may not be produced with the same standardization and rigor as research data; as such, some concern exists about their re-use for secondary purposes [7]. Therefore, investigations into data quality are necessary to determine whether the data are ‘fit for purpose’. Previous studies evaluating the quality of primary care EMR data in Canada have typically reported on a limited aspect of quality (e.g. completeness) or data elements [8–10] or have assessed quality more broadly without focusing on a specific context for use [11, 12]. The objective of this study was to comprehensively assess the quality of primary care EMR data in Alberta, Canada within the context of hypertension.

## Methods

### Data source

The Canadian Primary Care Sentinel Surveillance Network (CPCSSN) is a collaboration of eleven practice-based research networks (PBRN) across Canada who manage the extraction, cleaning and processing of de-identified EMR data from primary care settings [13]. At present, over 1200 primary care providers and 1.8 million patients contribute data from eight provinces and territories [14]. National CPCSSN data have been previously used to report on the epidemiology of many conditions in primary care, such as hypertension [5], diabetes [15], depression [16], osteoarthritis [17], dementia [18], chronic obstructive pulmonary disease [19], and others. The CPCSSN organization and data extraction and processing have been described elsewhere [13, 20].

This data quality assessment utilized primary care EMR data obtained by the two PBRNs in the province of Alberta – the Northern and Southern Alberta Primary Care Research Networks (NAPCReN and SAPCReN, respectively). Because healthcare in Canada is organized and delivered separately within each province or territory, only one province (Alberta) was chosen for the data quality assessment in order to minimize variation in the data due to interprovincial differences such as healthcare delivery and practice, drug coverage, health information legislation, EMR uptake and extent of use, types of EMR systems available, and many other factors [21, 22].

In Alberta, there were 323 providers (mostly family physicians with a small proportion of nurse practitioners and community pediatricians) participating from 53 primary care practices. This represents slightly over 5% of the total number of family physicians in Alberta [23]. As of June 2018, de-identified EMR data were extracted from 397,518 patients in total; this reflected approximately 9.2% of Alberta’s general population of 4.3 million people [24]. The CPCSSN data has previously been found to overrepresent older adults and women [25], but this is typical of primary care populations.

Currently, CPCSSN in Alberta extracts from five distinct EMR systems – Wolf, Med Access, Practice Solutions Suite, Accuro and Healthquest. The earliest (or ‘start’) date of information in the CPCSSN database varies by clinic and by patient, depending on when a clinic first implemented their EMR system, as well as when the patient first attended the clinic.

### Patient sample

Adult patients (18 years and older) who had at least one primary care encounter in the previous two years (July 1, 2016 to June 30, 2018) were included, in order to establish an ‘active’ patient population. Any patient who was recorded as ‘deceased’ or ‘inactive’ in the EMR was excluded, as were any patients or providers who had explicitly requested to opt out of the CPCSSN database. The data quality assessment focused specifically on patients with hypertension who were identified using a CPCSSN-developed definition [26]. The hypertension definition consisted of a combination of International Classification of Disease version nine (ICD-9) codes (401, 402, 403, 404, 405) and medications located throughout the EMR: a minimum of two physician billing codes within two years *or* any occurrence of a diagnosis in the Problem List/Profile *or* prescription for an anti-hypertensive medication (with medication criteria alone being insufficient if other specific diagnoses exist, such as heart failure or diabetes) [26]. The definition was validated using chart reviews as the reference standard and demonstrated good sensitivity (84.9%) and specificity (93.5%) [26].

### Data quality assessment

The data quality assessment was a cross-sectional, descriptive evaluation guided by reporting recommendations for distributed data networks [27]. Data elements were selected based on their potential use and relevancy for hypertension surveillance, as well as availability in the CPCSSN data. These included: patient demographics; physical examinations (weight, height, body mass index [BMI], and systolic and diastolic blood pressure); laboratory values (high density lipoprotein [HDL] cholesterol, low density lipoprotein [LDL] cholesterol, total cholesterol, triglycerides, fasting blood glucose, glycated hemoglobin [HbA1C]), anti-hypertensive medications (defined using categories of the relevant groups of Anatomical Therapeutic Chemical [ATC] codes: C02*, C03*, C07*, C08*, C09*); and risk factor records for smoking and alcohol use. Only the CPCSSN-processed/coded values were used, as these are typically the data elements that are accessible from CPCSSN for secondary purposes. A full description of all data elements can be found in the CPCSSN Data Dictionary online [14].

Summary statistics were reported for continuous variables, which included range, mean, and median. Proportions (restricted to the three most frequent values) and number of unique values were described for categorical variables. Missingness was reported as a proportion of patients without a recorded data element (e.g. height) or record (e.g. medication, smoking); missingness of specific items within a record was also reported (e.g. dose in medication record). Data completeness was also represented visually by clinic and EMR type.

Several temporal aspects of the data were examined – the proportion of patients who had at least one physical exam measurement or laboratory value documented in the previous year (July 1, 2017 to June 30, 2018) was reported, in addition to the proportion of risk factor (i.e. smoking and alcohol) and medication records that contained a stop/end date prior to the start date. An exploration of patient-level weight values over time were visualized by plotting the difference between subsequent weight measurements and the length of time (days) between subsequent measurements for individuals with at least two weight measurements.

External validity was evaluated by comparing the most recent crude hypertension prevalence estimates from three national population-level sources: administrative data from the Canadian Chronic Disease Surveillance System (CCDSS), consisting of physician billing claims, hospitalizations and prescription drug records [28]; the Canadian Health Measures Survey (CHMS), which defines hypertension based on standardized, direct BP measurements and health-related interviews [29]; and self-reported high BP from the Canadian Community Health Survey (CCHS) [30]. Hypertension prevalence estimates from the national CPCSSN data [5] were also used as a comparison to the regional-level (Alberta) data.

RStudio version 1.1.456 was used for the analysis, which was conducted in 2019. This study was approved by the University of Calgary’s Conjoint Health Research Ethics Board (REB17–1825) and the University of Alberta’s Health Research Ethics Board (Pro00079372).

## Results

**Fig. 1:**
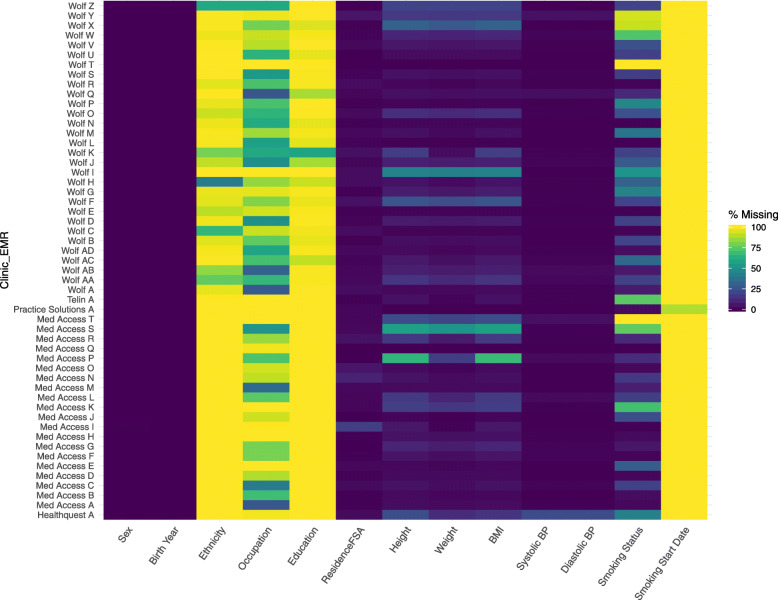
Summary of Completeness of Select EMR Data Elements by EMR Type and Clinic for Patients with Hypertension

In the CPCSSN data for Alberta, there were 205,364 adult patients who had at least one primary care encounter in the previous two years; of these, 48,377 patients were identified with hypertension and who were not labelled ‘inactive’ at the practice or deceased. Patients in the hypertension sample had a median of 8.0 years (IQR 7) of information in their record. Figure 1 provides a visual summary of the completeness of data for patient demographics, physical measurements, and smoking status by each of the 53 clinics and 5 EMR systems included in the data quality assessment. The data element characterization in Tables 1, 2, 3 and 4 provides a more in-depth examination of the quality of hypertension-related variables.

**Table 1:** Missingness and summary statistics for patient demographic information^a^

Data element	Summary	Males *N* = 23,486 (48.5%)	Females *N* = 24,889 (51.5%)
Year of birth	No. patients missing, n (%)	0 (0)	0 (0)
	Range	1915–1998	1914–1998
	Mean (SD)	1954 (13.4)	1952 (14.4)
	Median (IQR)	1954 (17)	1951 (19)
Ethnicity	No. patients missing, n (%)	22,511 (95.8)	23,788 (95.6)
	Unique values, n	76	83
	3 most frequent values, n (%)	“Caucasian”: 623 (63.9) “Aboriginal”: 103 (10.6) “Canadian”: 64 (6.6)	“Caucasian”: 588 (53.4) “Aboriginal”: 145 (13.2) “Canadian”: 99 (9.0)
Occupation	No. patients missing, n (%)	16,810 (71.6)	18,423 (74.0)
	Unique Values, n	3628	3536
	3 most frequent values, n (%)	“Retired”: 1080 (16.2) “Truck driver”: 125 (1.9) “Farmer”: 103 (1.5)	“Retired”: 1250 (19.3) “Homemaker”: 81 (1.3) “Teacher”: 80 (1.2)
Education	No. patients missing, n (%)	22,866 (97.4)	24,262 (97.5)
	Unique Values, n	10	12
	3 most frequent values, n (%)	“University”: 184 (22.7) “High School”: 176 (28.4) “College”: 89 (14.4)	“University”: 188 (30.0) “High School”: 175 (27.9) “College”: 151 (24.1)

*IQR* interquartile range, *SD* standard deviation

^a^Note: sex was missing for 2 patients

**Table 2:** Missingness and summary statistics for physical measurements and laboratory values

Data element	Summary	Males *N* = 23,486	Females *N* = 24,889
Height (cm)	No. patients missing, n (%)	2391 (10.2)	2345 (9.4)
	Patients with a measurement in previous year, n (%)	11,604 (49.4)	12,562 (50.5)
	Range	41.9–229.6	37.2–229.0
	Mean (SD)	174.6 (8.2)	160.8 (7.5)
	Median (IQR)	175.0 (10.0)	161.0 (9.2)
	Median number of total measurements per patient, n	4.0	5.0
Weight (kg)	No. patients missing, n (%)	1791 (7.6)	1714 (6.9)
	Patients with a measurement in previous year, n (%)	12,165 (51.8)	13,241 (53.2)
	Range	1.0–500.0	1.8–477.0
	Mean (SD)	104.9 (41.5)	90.4 (39.0)
	Median (IQR)	94.0 (30.3)	80.0 (33.0)
	Median number of total measurements per patient, n	4.0	5.0
BMI (kg/m^2^)	No. patients missing, n (%)	2422 (10.3)	2316 (9.3)
	Patients with a measurement in previous year, n (%)	11,979 (51.0)	13,062 (52.5)
	Range	5.1–199.9	5.0–200.0
	Mean (SD)	31.8 (9.5)	32.2 (11.1)
	Median (IQR)	30.2 (7.4)	30.1 (10.1)
	Median number of total measurements per patient, n	4.0	5.0
Systolic blood pressure (mmHg)	No. patients missing, n (%)	226 (1.0)	264 (1.1)
	Patients with a measurement in previous year, n (%)	19,898 (84.7)	21,069 (84.6)
	Range	52.0–266.0	54.0–290.0
	Mean (SD)	134.4 (17.2)	134.6 (17.7)
	Median (IQR)	133.0 (22.0)	133.0 (23.0)
	Median number of total measurements per patient, n	16.0	18.0
Diastolic blood pressure (mmHg)	No. patients missing, n (%)	226 (1.0)	264 (1.1)
	Patients with a measurement in previous year, n (%)	19,898 (84.7)	21,069 (84.6)
	Range	30.0–188.0	30.0–200.0
	Mean (SD)	80.4 (11.5)	78.8 (11.0)
	Median (IQR)	80.0 (16.0)	80.0 (15.0)
	Median number of total measurements per patient, n	16.0	18.0
Fasting glucose (mmol/L)	No. patients missing, n (%)	5005 (21.3)	5223 (21.0)
	Patients with a measurement in previous year, n (%)	2969 (12.6)	2826 (11.4)
	Range	1.6–42.9	1.5–35.0
	Mean (SD)	6.4 (2.0)	6.0 (1.8)
	Median (IQR)	5.7 (1.5)	5.5 (1.2)
	Median number of total measurements per patient, n	4.0	4.0
Hemoglobin A1C (%)	No. patients missing, n (%)	2905 (12.4)	3283 (13.2)
	Patients with a measurement in previous year, n (%)	12,471 (53.1)	12,305 (49.4)
	Range	3.1–18.6	1.0–18.2
	Mean (SD)	6.7 (1.4)	6.6 (1.3)
	Median (IQR)	6.3 (1.6)	6.2 (1.3)
	Median number of total measurements per patient, n	4.0	3.0
Low-density lipoprotein (LDL) cholesterol (mmol/L)	No. patients missing, n (%)	1888 (8.0)	2343 (9.4)
	Patients with a measurement in previous year, n (%)	9429 (40.1)	9249 (37.2)
	Range	0.0–9.8	0.0–9.5
	Mean (SD)	2.5 (1.0)	2.8 (1.0)
	Median (IQR)	2.4 (1.4)	2.7 (1.3)
	Median number of total measurements per patient, n	5.5	4.0
High-density lipoprotein (HDL) cholesterol (mmol/L)	No. patients missing, n (%)	1725 (7.3)	2277 (9.1)
	Patients with a measurement in previous year, n (%)	9616 (40.9)	9294 (37.3)
	Range	0.0–6.5	0.2–7.0
	Mean (SD)	1.2 (0.3)	1.5 (0.4)
	Median (IQR)	1.1 (0.4)	1.4 (0.5)
	Median number of total measurements per patient, n	4.0	4.0
Total cholesterol (mmol/L)	No. patients missing, n (%)	1714 (7.3)	2233 (9.0)
	Patients with a measurement in previous year, n (%)	9659 (41.1)	9338 (37.5)
	Range	0.7–23.3	0.6–21.5
	Mean (SD)	4.5 (1.1)	5.0 (1.1)
	Median (IQR)	4.4 (1.6)	4.9 (1.5)
	Median number of total measurements per patient, n	5.0	5.0
Triglycerides (mmol/L)	No. patients missing, n (%)	1713 (7.3)	2261 (9.1)
	Patients with a measurement in previous year, n (%)	9771 (41.6)	9414 (37.8)
	Range	0.2–29.6	0.2–29.9
	Mean (SD)	1.8 (1.3)	1.7 (1.0)
	Median (IQR)	1.5 (1.1)	1.5 (1.0)
	Median number of total measurements per patient, n	5.0	4.0

*BMI* body mass index, *cm* centimetres, *HDL* high-density lipoprotein, *IQR* interquartile range, *kg* kilograms, *LDL* low-density lipoprotein, *mmHg* millimeter of mercury, *mmol/L* millimoles per litre, *SD* standard deviation

**Table 3:** Missingness and summary statistics for anti-hypertensive medications

Data element	Summary	Males *N* = 23,486	Females *N* = 24,889
Anti-hypertensive medication	No. patients with prescription at any time, n (%)	21,544 (91.7)	23,184 (93.1)
	Mean prescriptions per patient, n (SD)	12.1 (18.0)	12.0 (17.4)
	Median prescriptions per patient, n (IQR)	6.0 (10)	6.0 (11)
Medication name	No. records missing med name, n (%)	0 (0)	0 (0)
	Unique values, n	86	87
	3 most frequent values, n (%)	‘Ramipril’: 33,109 (12.7) ‘Amlodipine’: 27,772 (10.7) ‘Hydrochlorothiazide’: 26,238 (10.1)	‘Hydrochlorothiazide’: 40,537 (14.6) ‘Amlodipine’: 26,892 (9.7) ‘Ramipril’: 25,721 (9.3)
Start date	No. records missing start date, n (%)	0 (0)	0 (0)
	Unique values, n	5471	5604
Stop date	No. records missing stop date, n (%)	34,404 (13.2)	39,686 (14.3)
	Unique values, n	5664	5751
	Stop date occurs before start date, n (%)	2272 (0.9)	2364 (0.9)
Drug identification number (DIN)	No. records missing DIN, n (%)	135,564 (52.1)	150,923 (54.3)
	Unique values, n	1175	1238
	3 most frequent values, n (%)	‘326844’: 5264 (4.2) ‘878928’: 5262 (4.2) ‘2123282’: 4324 (3.5)	‘326844’: 7091 (5.6) ‘878928’: 6265 (4.9) ‘2123282’: 3452 (2.7)
Strength	No. records missing strength, n (%)	22,914 (8.8)	32,396 (11.7)
	Unique Values, n	95	99
	Median (IQR)	12 (35)	20 (35)
Dose	No. records missing dose, n (%)	5428 (2.1)	7387 (2.7)
	Unique values, n	2048	2730
	3 most frequent values, n (%)	‘1’: 202,129 (79.3) ‘0.5’: 15,563 (6.1) ‘2’: 8635 (3.4)	‘1’: 208,017 (77.0) ‘0.5’: 18,644 (6.9) ‘2’: 7829 (2.9)
Frequency	No. records missing frequency, n (%)	24,013 (9.2)	33,261 (12.0)
	Unique values, n	133	117
	3 most frequent values, n (%)	‘QD’: 140,843 (59.6) ‘OD’: 45,494 (19.3) ‘BID’: 32,140 (13.6)	‘QD’: 145,261 (59.5) ‘OD’: 52,094 (21.3) ‘BID’: 27,764 (11.4)
Duration count	No. records missing duration, n (%)	33,013 (12.7)	38,379 (13.8)
	Unique values, n	3507	3692
	3 most frequent values, n (%)	‘3’: 51,704 (22.8) ‘100’: 50,550 (22.2) ‘90’: 21,444 (9.4)	‘3’: 51,928 (21.7) ‘100’: 49,245 (20.6) ‘90’: 20,498 (8.6)
Duration unit	No. records missing duration unit, n (%)	39,628 (15.2)	47,796 (17.2)
	Unique values, n	5	5
	3 most frequent values, n (%)	‘Day’: 139,344 (63.2) ‘Month’: 72,838 (33.0) ‘Week’: 5893 (2.7)	‘Day’: 145,621 (63.4) ‘Month’: 77,349 (33.7) ‘Week’: 4025 (1.8)
Dispensed count	No. records missing dispensed count, n (%)	28,376 (10.9)	33,779 (12.2)
	Unique values, n	3688	3732
	Median (IQR)	100 (90)	91 (95)
Dispensed form	No. records missing dispensed form, n (%)	74,277 (28.5)	93,218 (33.6)
	Unique values, n	3	5
	3 most frequent values, n (%)	‘Tab’: 158,009 (84.9) ‘Capsule’: 27,994 (15.1) ‘Bottle’: 1 (0)	‘Tab’: 161,436 (87.6) ‘Capsule’: 22,859 (12.4) ‘Bottle’: 3 (0)
Reason for medication	No. records missing reason, n (%)	199,924 (76.8)	210,112 (75.7)
	Unique values, n	281	322
	3 most frequent values, n (%)	‘Hypertension, CHF’: 21,278 (35.3) ‘Hypertension, Angina’: 11,743 (19.5) ‘Hypertension’: 7711 (12.8)	‘Hypertension, CHF’: 20,928 (31.0) ‘Hypertension, Angina’: 12,268 (18.2) ‘Hypertension’: 10,337 (15.3)

*IQR* interquartile range, *SD* standard deviation

**Table 4:** Missingness and summary statistics for risk factor records

Data element	Summary	Males *N* = 23,486	Females *N* = 24,889
Smoking status	No. patients missing smoking record, n (%)	4716 (20.1)	5413 (21.7)
	Unique values, n	4	4
	3 most frequent values in all records, n (%)	“Unknown”: 17,942 (33.8) “Never”: 16,760 (31.6) “Current”: 11,799 (22.2)	“Never”: 20,018 (38.6) “Unknown”: 18,812 (36.3) “Current”: 7658 (14.8)
	Mean number of smoking records per patient, n (SD)	2.8 (4.2)	2.7 (3.6)
	Median number of smoking records per patient, n (IQR)	1.0 (2.0)	1.0 (2.0)
Smoking start date	No. records missing start date, n (%)	52,999 (99.9)	51,780 (99.9)
	Smoking end date occurs before start date, n (%)	0 (0)	0 (0)
Alcohol use status	No. patients missing alcohol record, n (%)	12,372 (52.7)	15,034 (60.4)
	Unique values, n	5	7
	3 most frequent values in all records, n (%)	‘Current’: 7228 (93.6) ‘Past’: 479 (6.2) ‘Unknown’: 11 (0.1)	‘Current’: 6068 (94.8) ‘Past’: 220 (3.4) ‘Unknown’: 108 (1.7)
	Mean number of alcohol records per patient, n (SD)	2.0 (2.2)	2.2 (2.6)
	Median number of alcohol records per patient, n	1.0 (1.0)	1.0 (1.0)
Alcohol use start date	No. records missing start date, n (%)	21,939 (100)	22,127 (100)
	Alcohol use end date occurs before start date, n (%)	0 (0)	0 (0)

### Patient demographics

Birth year was complete for all patients, as was sex (with the exception of two patients). However, nearly all socio-demographic information on patients was mostly incomplete (Table 1). For those who had some information recorded in ethnicity, occupation, or education fields, the data were highly inconsistent – for instance, over 3500 unique entries were recorded for occupation and more than 75 distinct entries were found for ethnicity.

### Height, weight, BMI

**Fig. 2:**
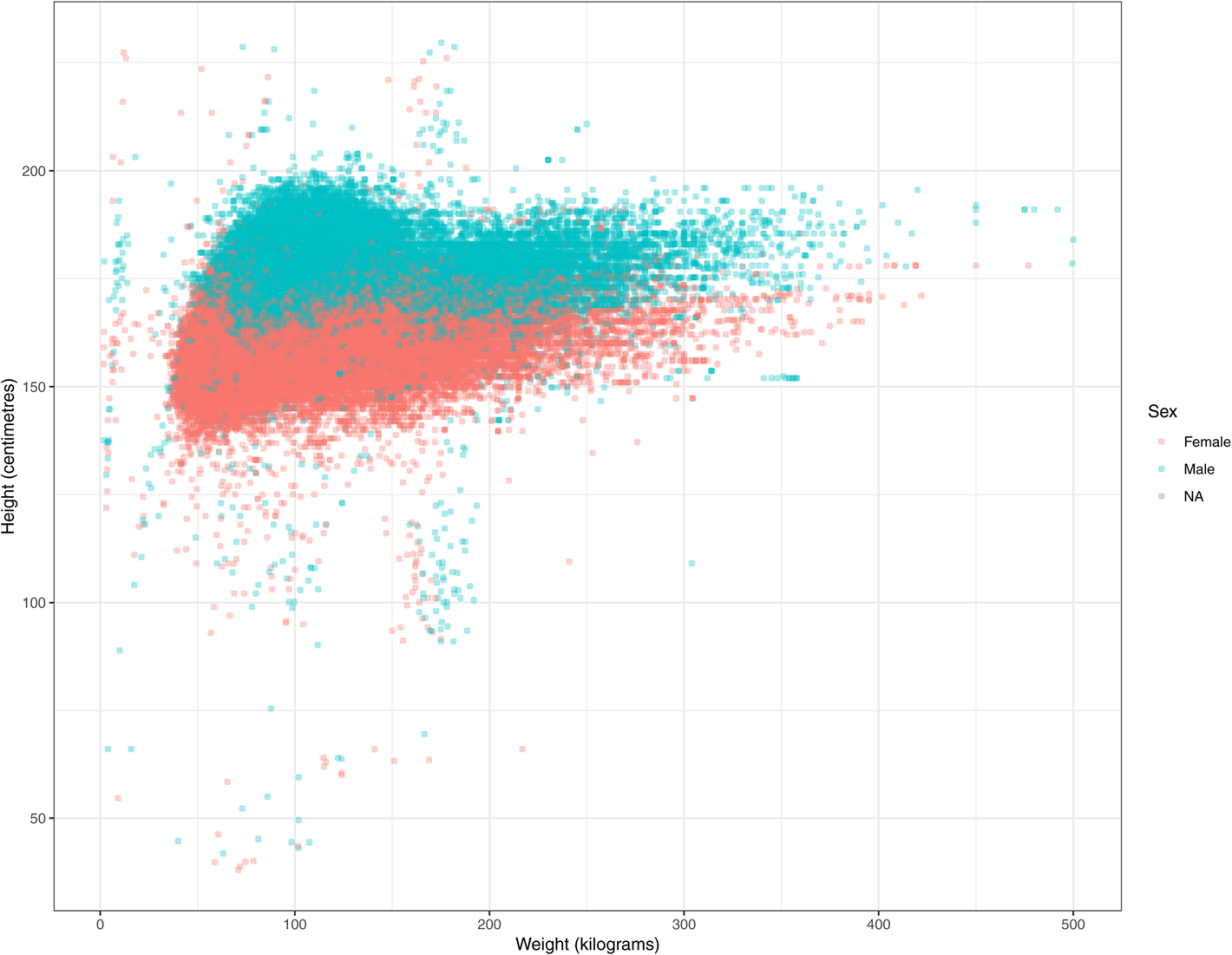
Paired Height and Weight Measurements in Patients with Hypertension

Approximately 10% of patients were missing a height or BMI value and even fewer patients were missing weight (Table 2). Males had a median of four measurements for height, weight, or BMI and females had five, with these measurements showing a skewed distribution. From the lower and upper ranges of the height, weight and BMI values, it appears that data errors are present. For example, female weight values ranged from 1.8 to 477 kg, which is biologically unlikely. When plotting patient-level height and weight values (Fig. 2), those located outside the main cluster of points visually identify specific data errors. For instance, the vertical line of points approaching 0 on the x (weight) axis might indicate a data entry error (e.g. weight entered as 10 instead of 100) or swapped height and weight values (e.g. height in metres entered in the weight field). Another observable area of atypical points was between 150 and 200 on the x (weight) axis, which potentially represents height and weight values that were entered in the wrong fields (e.g. weight = 175 and height = 100 recorded instead of weight = 100 kg and height = 175 cm).

**Fig. 3:**
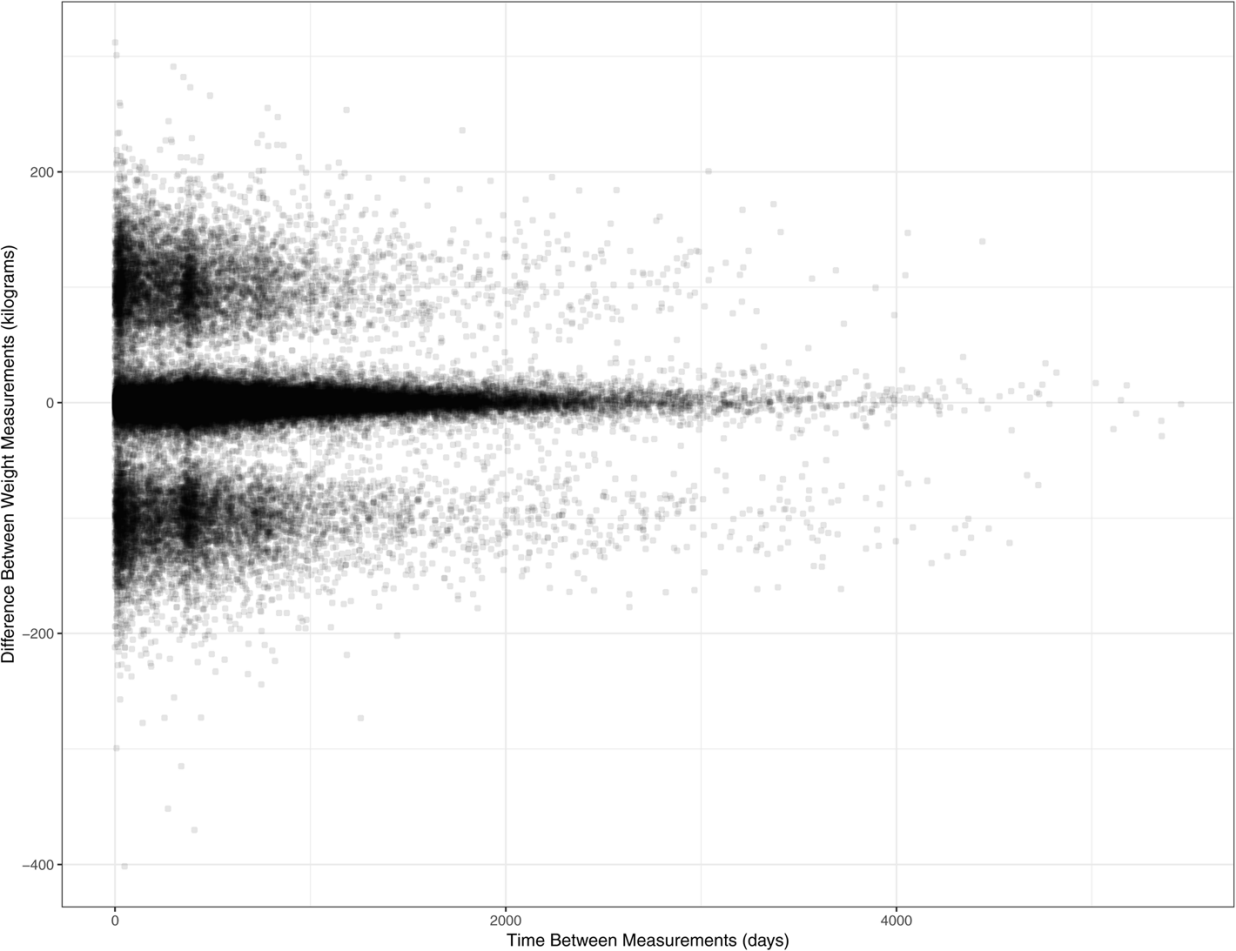
Differences in Subsequent Patient Weight Measurements Throughout Time in Patients with Hypertension. Note: patients with two or more weight values recorded at any time in their EMR (*n* = 39,202)

Figure 3 investigates possible errors in weight values using successive patient-level weight measurements for those who had at least two weight values recorded in their EMR (*n* = 39,202). It would be expected that changes in individual weight might demonstrate more variability over time (e.g. patient weight recorded 10 days apart should have minimal difference, whereas weight measurements taken several years apart might show a more significant change). Two peaks centred around 100 and − 100 on the y-axis emerged as potentially problematic data: in a relatively short time period between measurements, the difference between successive weight measurements was approximately 100 kg for patients clustered around those two peaks. This likely represents inconsistencies in the unit of measurement (e.g. kilograms versus pounds) for subsequent weight measurements for a given individual. However, the extent of the problem was not substantial – the majority of weight values (94.8%) occurred within two standard deviations of the central peak (mean − 0.29) and at least one potential data error (i.e. outside two standard deviations) at any time was detected in the records of 18.4% of patients.

### Blood pressure

BP measurements were well-recorded in terms of completeness (99%) and the majority of patients (85%) had at least one measurement recorded in the previous year (Table 2). However, BP values at the minimum and maximum end of the range may indicate data errors (Table 2). These values could be biologically possible, but would be very unlikely in an outpatient setting; for instance, a systolic BP of 52 might indicate shock and a systolic BP of 290 would be an emergency event. In addition, CPCSSN also sets limits to BP values when processing the raw EMR data (50–300 mmHg for sBP; 20–200 mmHg for dBP), which would underestimate the true range of values.

Male patients had a median of 16 total BP measurements recorded in their EMR and females had slightly more (median = 18). A small proportion of patients had large sums of annual BP measurements – for instance, 4.2% of females and 4.0% of males were above the 95th percentile for number of BP measurements (greater than 10) in 2017 (data not shown).

### Laboratory values

Of the laboratory tests measuring blood glucose, HbA1C values were present in the EMR more often than fasting glucose (88% versus 79% of patients), and more patients had an HbA1C test result in the previous year compared to the fasting glucose test (Table 2).

The lipid values included in this assessment (LDL, HDL, total cholesterol, triglycerides) were available for the majority of patients in this cohort (at least 91%, varying by lab type), with a median of 4–5 values for each patient in the EMR (Table 2). Female patients were observed to have a slightly fewer lipid values present in their EMR compared to male patients (Table 2).

For all types of lab results, the upper and lower limits were unlikely to be seen in an outpatient setting (i.e. primary care) and many values were beyond a biologically plausible range (e.g. HDL and LDL lower value = 0). This points to likely data errors at the upper and lower ends of the range of values, however, it was only for a very small proportion of lab values.

### Hypertensive medications

The vast majority of males (92%) and females (93%) with hypertension had at least one recorded anti-hypertensive prescription, with a median of six anti-hypertensive medication prescriptions per person (Table 3). The medication records themselves were fairly complete; all records contained a start date and most contained a stop date, strength, dose, frequency, duration, and count. Drug Identification Number (DIN) and ‘reason for medication’ mostly incomplete, with DIN missing in over half of medication records and ‘reason’ missing in over three-quarters of records.

### Smoking and alcohol status

Within the Risk Factor section in the EMR, nearly 80% of patients had a smoking status recorded, with ‘Unknown’ and ‘Never’ as the most frequently recorded categories (Table 4). However, after excluding the indiscriminate ‘Unknown’ smoking status, a total of 31,976 patients (66.1%) and 68,110 records remained across three categories: ‘Current’, ‘Past’ or ‘Never’ (data not shown). Males and females had a similar number of smoking records per person (median = 1; mean = 3). All start and end dates were missing from the records.

More males than females had their alcohol use recorded (47 and 40%, respectively) and these records were primarily for ‘Current’ users (Table 4), indicating that alcohol use is likely recorded differentially between users and non-users. Patients had a mean of 2 records in their EMR (median of 1) and no records contained start or end dates.

Of note, a ‘Date Created’ field exists for both smoking and alcohol records. This field indicates when the record was created in the EMR system but does not necessarily correspond to the start of the risk behaviour. ‘Date Created’ was present in 80.6% of smoking records and 76.6% of alcohol use records.

### External validity

The overall crude estimate for Alberta-specific hypertension prevalence in the CPCSSN data (23.6%) were similar to the 2014–15 physical measure survey (CHMS) (23.3%) and was also comparable to the national CPCSSN estimate (22.8%) (Table 5). The largest discrepancy was seen in the self-reported CCHS, with hypertension prevalence estimated at 17.7%. Male patients in the CPCSSN database had a higher hypertension prevalence (26.1%) than all other sources, while the prevalence for female patients (21.6) in the CPCSSN data was similar to the health measures survey (22.0%) and slightly lower than the CCDSS (25.6%).

**Table 5:** Prevalence comparison of adults with hypertension in various data sources

Data source	Data source type	Year	Sample	Overall (crude %)	Males (crude %)	Females (crude %)
CPCSSN (Alberta-specific)	Primary care EMR data	2018	Albertans 18 years & older	23.6	26.1	21.6
National CPCSSN data [5]	Primary care EMR data	2012	Canadians 18 years & older	22.8	24.1	21.9
CCDSS [28]	Administrative data	2015	Canadians 20 years and older	25.4	25.2	25.6
CHMS [29]	Physical measures survey	2014–15	Canadians 20–79 years	23.3	24.3	22.0
CCHS [30]	Self-reported survey	2016	Canadians 12 years and older	17.7	18.3	17.1

*CCDSS* Canadian Chronic Disease Surveillance System, *CCHS* Canadian Community Health Survey, *CHMS* Canadian Health Measures Survey, *CPCSSN* Canadian Primary Care Sentinel Surveillance Network, *EMR* Electronic medical record

## Discussion

This paper describes the quality of primary care EMR data in Alberta within the context of utilization for hypertension surveillance and epidemiology. Overall, there was observable variability due to the type of EMR system, between clinics, and among the data elements themselves. As this assessment focused on patients with hypertension, it was not surprising to see blood pressures and prescribed medication records that were largely complete and contained minimal outliers; these data constitute a particularly valuable contribution for surveillance purposes, given that BP and prescribing information are not available in administrative data or are limited (i.e. cross-sectional) in survey data. Although these data cannot confirm whether a patient has filled their prescription or is adherent, the information within the medication records are relatively complete and can be used to approximate persistence/adherence, for example, by calculating medication possession ratio or using similar methods [31].

The select laboratory values were present in the EMR of the majority of patients in this cohort, with the exception of fasting blood glucose. This aligns with current clinical guidelines recommending routine testing of lipids and blood glucose/glycated hemoglobin for individuals with hypertension [32]. Although most laboratory test results in Alberta are imported directly into the EMR from the community lab provider, data quality issues were still present, although to a very small degree. The observed range of values demonstrated upper and lower limits that are not likely in an outpatient setting and some that were biologically implausible (e.g. 0 mmol/L for LDL and HDL; 43 mmol/L for fasting glucose). These errors may have been introduced during the import of lab results to the EMR or during the CPCSSN processing to convert different units of measurement to a standard unit (e.g. mmol/mol to % for HbA1C).

Other information, such as sociodemographic, height, weight, and risk factor information, were more inconsistent and less complete. Although achieving 100% completeness for all data elements may not be realistic, it is not unreasonable to aim for near complete information for these data elements at the point of care. Cardiovascular disease guidelines suggest that smoking status should be updated on a regular basis and given that screening is often risk based, information about alcohol use, height, weight, BMI, and ethnicity are particularly important to document for a hypertensive cohort [32]. However, distinguishing between data that are missing due to inadequate data entry or as a result of not extracting the data is difficult. One significant challenge when addressing poor data quality is determining the source of the issue – for instance, missing data may be due to the unavailability of these fields in certain EMR systems (in which case, missingness will always exist); patients might not be asked about specific topics, such as alcohol use or ethnicity, or they may decline to answer; lastly, the CPCSSN processes may omit extraction from certain fields of the EMR either deliberately (e.g. identifiable fields or physician notes) or unintentionally (e.g. if an EMR system upgrade changes the names of data elements, which would subsequently affect the CPCSSN extraction code). Identifying true inaccuracies in the data are similarly problematic; this may be possible for some data elements through a chart review, with particular attention to the detailed physician notes and scanned documents (i.e. specialist letters, diagnostic imaging) that are not currently captured in the CPCSSN data. However, this is a time-intensive method and the structured EMR fields are likely to contain the same errors and omissions as the CPCSSN data. Beyond this, confirming with or measuring patients directly to verify data elements in the EMR would most accurately reveal true data errors but this method is also the least feasible.

Therefore, the most appropriate strategies for preventing and mitigating EMR data quality issues should be multifaceted and involve a variety of settings. CPCSSN has largely taken a post-extraction analytic approach to data improvement. This includes extensive cleaning and coding algorithms, the development and validation of case definitions for various conditions that are made availble as part of the database [26, 33–35], and exploration of more advanced techniques like natural language processing [36] and machine learning [35]. As an example, CPCSSN is currently developing a pattern-matching algorithm that aims to enhance the completeness and accuracy of smoking records. In the raw or original EMR data, some additional information related to smoking, such as frequency of tobacco use and quantity of tobacco units consumed (e.g. cigarettes / cigars, packs), is present but primarily in unstructured, lengthy text strings that is not useful for analysis, may also contain identifiable patient information, and is therefore not currently available to researchers. The pattern-matching algorithm is designed to extract only smoking-related information from the free text and categorize the record into a defined smoking status, leading to more available coded data for researchers to access.

A number of other strategies have been shown to improve the completeness and accuracy of EMR data – some occur at the practice level, such as employing a dedicated data entry clerk [37] or providing data quality audit and feedback reports to clinicians [38]. Other initiatives require more substantial resources and uptake, such as mandated national EMR content standards [39], developing EMR interfaces that are easier to navigate and contain more stuctured fields, and promoting financial or other incentives for ‘meaningful EMR use’ [40].

In the future, routine linkage to other data sources, like administrative health data, could enhance quality by providing a mechanism to verify certain aspects of EMR data and expand the breadth of information about individual patients throughout the broader healthcare system.

### Limitations

This paper provides a quality assessment of select CPCSSN data elements deemed to be important for hypertension surveillance or research, but it was not possible to examine and report on all variables contained in the CPCSSN database in a single manuscript, nor was it possible to examine discrete cardiovascular outcomes related to hypertension (for example, hospitalization for myocardial infarction), as this information is usually contained in other databases external to CPCSSN or captured in the EMR in an inaccessible format (e.g. PDF document, free text notes). It was also not feasible to quantify the true accuracy of data elements, other than appraising the plausibility of values through descriptive means. Secondly, during the CPCSSN processing and data transformation stages for physical exam measurements and some lab types, restrictions are introduced for out-of-bounds values and thus, the summary statistics presented in this paper may not reflect the full variation of values originating from the source EMR. In addition, any changes or improvements made to the CPCSSN processing may result in slight differences in the CPCSSN EMR database between each extraction cycle. Thirdly, although the CPCSSN definition for hypertension demonstrated high sensitivity and specificity, a potential for misclassification still exists. This may have underestimated the number of patients with hypertension or produced a patient sample that is biased towards a greater severity of illness. Lastly, the quality was described specifically for CPCSSN data from Alberta and within the context of hypertension. This is not a population-level data source and only constitutes a sample of participating providers and patients who have sought care. Thus, the overall findings may not be representative of the wider Alberta population or for other provinces or territories that participate in CPCSSN, and may also differ in other disease-based contexts. However, CPCSSN has developed uniform data extraction, processing, and standardization methods across the country, which may allow for other regional networks to compute the same data quality assessment for comparison.

## Conclusion

Primary care EMR data are a valuable data source for hypertension surveillance or within an epidemiological context. The high-quality and longitudinal blood pressure and prescribed antihypertension medication data are particularly useful, as these types of data are not found in traditional administrative databases. Other data elements, such as sociodemographics, physical examination values, laboratory results, and risk factor information, exhibited variation in quality. These data elements may be less useful in their current state but offer promising value in the future once data quality issues can be addressed through additional pre- or post-extraction solutions.

## Data Availability

The national CPCSSN data are available to approved researchers for a fee; for more information or to submit a Letter of Intent, visit: http://cpcssn.ca/research-resources/. The Alberta-specific CPCSSN data that was used for this analysis are available as two separate data sets through the regional networks (NAPCReN, SAPCReN). Data access procedures and requirements vary by network; contact the corresponding author for more information or visit: http://napcren.ca or http://sapcren.ca

## Abbreviations

ATC: Anatomical Therapeutic Chemical [classification system]
BMI: Body mass index
BP: Blood pressure
CCDSS: Canadian Chronic Disease Surveillance System
CHMS: Canadian Health Measures Survey
cm: Centimetres
CPCSSN: Canadian Primary Care Sentinel Surveillance Network
dBP: Diastolic blood pressure
DIN: Drug Identification Number
EMR: Electronic medical record
HbA1C: Hemoglobin A1C (glycated hemoglobin)
HDL: High density lipoprotein
ICD-9: International Classification of Disease version 9
IQR: Interquartile range
kg: Kilogram
LDL: Low density lipoprotein
mmHg: Millimetres mercury
mmol/L: Millimoles per litre
NAPCReN: Northern Alberta Primary Care Research Network
PBRN: Practice-based research network
SAPCReN: Southern Alberta Primary Care Research Network
sBP: Systolic blood pressure
SD: Standard deviation
